# 

**Published:** 1882-05

**Authors:** 


### AMERICAN DENTAL ASSOCIATION.

The annual meeting of the American Dental Association will be held in Cincinnati August 1st to the 4th inclusive. Arrangements are being made such as cannot be otherwise than acceptable and satisfactory to all interested. The sessions will probably be held above the tumult and heat of the city. It can now be said that a large reduction on hotel rates will be made, and it is expected that the railroads will make liberal reductions on rates.

Let no State or local society fail to appoint the full quoto of delegates, and let none, who are appointed, fail in coming. All the prominent members, of course, will come. The delegates and permanent members should bring their wives or daughters. In addition to all this, it is very desirable to have a large number of visitors, who will, by their presence, encourage the members, and will themselves become interested and enthused, and return home, join their local society and return next year as delegates.

Let the members and delegates be liberal in their invitations to visitors.

In the May number the arrangements will be more explicitly given.

INTERNATIONAL MEDICAL CONGRESS.—It has been decided that the next International Medical Congress will be held in Copenhagen, in 1884.

A DEATH from chloroform is reported as having recently occurred at Munich, in the case of a woman on whom a dental operation was being performed.

### ILLINOIS STATE DENTAL SOCIETY.

The eighteenth annual meeting of the Illinois State Dental Association will be held at Quincy, beginning Tuesday, May 9, 1882, and continue four days. Preparations have been made for a grand good meeting, as may well be inferred from the following:

Clinics will be held through one morning only. They will be conducted by ten operators, who will enable persons interested to see many operations in a short time.

Drs. Pollock and Taggart will opperate with the electric magnetic mallet.

Dr. Allport will demonstrate his method of operating with non-cohesive gold.

Drs. Morrison and Patrick will demonstrate new and improved plans for the construction and application of artificial crowns to the natural roots.

Dr. Patrick will exhibit a new and simple device for correcting irregularities of the teeth, and will deliver a lecture Wednesday evening upon "The Mechanism of the Jaws of the Mammalia," illustrated.

Dr. Haskell will present an outfit for a model dental laboratory.

The following subjects will be presented and considered by papers and discussions, viz.:

1st. Mechanical Dentistry.
2d. Random Thoughts from the Laboratory.
3d. Operative Dentistry.
4th. Materials and Methods for Preserving the Teeth.
5th. Caries and Necrosis of the Maxillary Bones.
6th. The So-called Riggs Disease.
7th. Indications for, and Best Methods of Extracting Teeth.
8th. Artificial Crowns.
9th. High Civilization Not the Cause of Tooth Decay.
10th. Dental Education.
11th. Quinine, Its Use in Dentistry.
12th. Periodontitis, Cause and Treatment.

With all these inducements it is difficult to see how any one can stay away, if possible to be present.

### NORTHERN OHIO DENTAL ASSOCIATION.

The twenty-third annual meeting of this body will be held in Cleveland at the rooms of the Dental Depot, 306 Euclid Avenue, on Tuesday and Wednesday, May 9th and 10th, 1882. Commencing at 10 o'clock A. M., on Tuesday.

The following subjects will be presented for consideration:

1st. Filling Teeth with Gold.

2d. Dental Prosthesis.

3d. Oral Surgery.

4th. Cases in Practice.

A cordial invitation is extended to all the profession to be present.

This is one of the substantial local societies of the country, and no member of the profession can attend its session without receiving benefit.

---

In the address of Dr. James Leslie, delivered recently before the graduating class of the Ohio College of Dental Surgery, and published in last number of the Register, the statement is made that the Dental Register was the first dental journal published.

The Doctor, in this statement, was under a misapprehension. The first number of the *American Journal of Dental Science* made its appearance in June, 1839. This was the first dental journal published in the world, so far as I am aware, and certainly in this country.

The first number of the Dental Register was issued October 1847, and has been issued regularly to the present time. The Register was the second dental journal in the United States, and is the oldest journal having a continuous issue. The *American Journal* was suspended some two years, or longer, after the death of Prof. Harris.

---

Professor Panum, of Copenhagen, has been chosen to preside over the next International Medical Congress, and Dr. Carl Lange has been appointed general Secretary.

### CONTENTS.

### COMMUNICATIONS.

### THE DENTAL REGISTER,

A MONTHLY MAGAZINE DEVOTED TO THE INTERESTS OF THE

**DENTAL PROFESSION.**

*Terms Reduced to* $2.00 *Per Year. Single Copies,* 25 *Cents.*

**EDITED BY PROF. J. TAFT, D.D.S.**

AND

***Published by SPENCER & CROCKER, Ohio Dental Depot.***

The volume commences in January and closes in December.

The REGISTER being issued monthly is always fresh, therefore Indispensable to the practitioner who desires to keep posted up with the new inventions and improvements which are daily developed. Neither expense nor effort will be spared to give value and variety to its contents. Articles will be illustrated with well executed engravings whenever they will add to the interest or assist to explanation.

Its extensive circulation and frequency of issue make it one of the BEST DENTAL ADVERTISING MEDIUMS in the United States.

All subscriptions must begin with January or July.

Local or special notices, five lines or less, will be inserted for $3 each insertion; over five lines, 50 cts. per line.

All advertising bills payable in advance, or at furthest, after the issue of the first number containing the advertisement. All transient advertisements to be paid for in advance.

☞ CONTRIBUTIONS SOLICITED.

Exclusively Editorial Communications should be addressed to the Editor.

The latest number of the REGISTER may be ordered with or without other goods at any time, and all letters should be addressed to

**SPENCER & CROCKER, Ohio Dental Depot, Cincinnati, Ohio.**

